# The Role of Biomaterials and Biocompatible Materials in Implant-Supported Dental Prosthesis

**DOI:** 10.1155/2021/3349433

**Published:** 2021-08-05

**Authors:** Reza Eftekhar Ashtiani, Mostafa Alam, Sara Tavakolizadeh, Kamyar Abbasi

**Affiliations:** ^1^Department of Prosthodontics, School of Dentistry, Shahid Beheshti University of Medical Sciences, Tehran, Iran; ^2^Department of Oral and Maxillofacial Surgery, School of Dentistry, Shahid Beheshti University of Medical Sciences, Tehran, Iran

## Abstract

The dental implant is one of the appropriate instances of the different dental materials and their application, which is the combined procedure of technology and science in physics, biomechanics, and surface chemistry from macroscale to nanoscale surface engineering and manufactured technologies. In recent decades, biomaterials in implant therapy promote bone response and biomechanical ability, which is long-term from surgical equipment to final prosthetic restoration. Biomaterials have a crucial role in rehabilitating the damaged structure of the tooth and supplying acceptable outcomes correlated with clinical performance. There are some challenges in implantation such as bleeding, mobility, peri-implant infections, and the solution associated with modern strategies which are regarded to biomaterials. Various materials have been known as promising candidates for coatings of dental implants which contain polyhydroxyalkanoates, calcium phosphate, carbon, bisphosphonates, hydroxyapatite, bone stimulating factors, bioactive glass, bioactive ceramics, collagen, chitosan, metal and their alloys, fluoride, and titanium/titanium nitride. It is pivotal that biomaterials should be biodegradable; for example, polyhydroxyalkanoates are biodegradable; also, they do not have bad effects on tissues and cells. Despite this, biomaterials have important roles in prosthetic conditions such as dental pulp regeneration, the healing process, and antibacterial and anti-inflammatory effects. In this review study, the role of biocompatible materials in dental implants is investigated in *in vitro* and *in vivo* studies.

## 1. Introduction

Nowadays, one of the dependable methods for repairing missing dentition is dental implant therapy which is broadly recognized. Bothe et al. in the 1940s explained the fundamental conception of metallic instrument implantation in bone for the first time. Then, Leventhal et al. represented that titanium has potential as a biocompatible material for surgical implantation 60 years ago [1]. The favorable outcome and purposes of the dental implants are crucially correlated with tissue response which is essential to find out the basic features of the tissue response to dental implantation [2]. Early failures of implants have been generally based on modified or inadequate wound healing which inhibits osseointegration [3]. Some other factors such as insufficient quality of bone, diversity of surgical technique, occlusal overload, and postoperative inflammation and infection have been involved in early failures of implantation. Delayed implant failures are usually the consequence of dissection in osseointegration, typically after the practical loading of implant-supported prostheses. Also, delayed failures of the implant have been commonly related to occlusal overload which is a biomechanical failure and peri-implantitis [4].

The restoring response to biocompatible materials is fibrous or fibrosis encapsulation. The repairmen after implantation generally include 2 procedures which are regeneration and replacement of connective tissue (contains fibrous capsule). The procedures are under the control of tissue framework persistence of implant location and cell proliferative capacity. The compatibility of dental implants is better with osseous tissue than soft tissue. Also, the bone-bonding power of dental implants is significant although the bonding characteristics with soft tissue are not acceptable and lead to the encapsulation of fibrous [2]. Many different types of materials are used in dentistry, which contains filling materials of intracanal, liners, medicaments of intracanal, subgingival implants, restorative materials, mouth rinses, and prosthetic materials [5]. As abundant dental devices and materials, vital necessities are resulting from systems of dental implants, since dental implant surface is straightly connected with critical soft/hard tissue and accustomed to chemical as well as mechanical features. At least, some necessities might involve morphological compatibility, mechanical compatibility, and biological compatibility to setting critical tissues [6]. Biomaterials have been also known as the biological or synthetic materials that are utilized to rehabilitate a segment of living structure to sustain involvement with tissues that are living. The systemic and local tissue responses state crucial characteristics of biocompatibility [2]. Furthermore, biomaterials containing dental implants and correlated components may be identified as any substances, synthetic or natural, that can be utilized for any time duration that deals with biological systems to improve the quality of life of each person of the society [7]. Any type of biocompatible materials can be prepared as nanoparticles that can beneficially enhance material properties in comparison with their same bulk ones. Also, research findings indicate that nanoparticles can be used as particle coatings to the surface of the dental implant to develop the integration of soft tissue and improve dental implant results [8]. After biomaterial implantation, the basic features of tissue response include acute and chronic inflammation, injury, interactions of blood material, formation of the provisional matrix, granulation tissue formation, progression of the fibrous capsule, and foreign body responses [2]. Classically, in biocompatibility terms, materials of bone graft are categorized as bioactive, biotolerant, or bioinert. Implant materials that are biotolerant are maintained in the body with encapsulation of fibrous due to the reaction of the tissue. Bioinert materials that are used as implants are associated with the bone tissue adjoining without chemical reactions. Bioactive implants set up chemical bonds with the bone tissue which causes straight deposition of bone matrix on the implant material. This theoretical categorization is established on histopathological observations of local influences after implantation into bone tissue [9]. Bioinert materials (e.g., stainless steel, stabilized zirconia, titanium, ultrahigh molecular weight polyethylene, and alumina) have minimum bonding with the encompassing tissue. Other materials have demonstrated the capability of biochemical and biophysical responses with around tissues. An actual chemical interaction capability with soft tissues has been displayed in several bioactive ceramics such as bioactive glasses of specific constitutions. Nevertheless, bioactive ceramics (bulk form) are inadvisable for load-bearing utilization because their strain-to-failure, the strength of flexuous, and toughness of fracture are less than those of bone, and their elasticity is more than that of bone [10]. Calcium phosphates ceramics are regarded to be osteoconductive and bioactive. The responses of ion exchange between the encompassing body fluids and bioactive implant set an active carbonate hydroxyapatite layer upon the implant that is the same as the mineral phase in bone. Bioresorbable bioactive materials also start to resorb upon the position of the human body and are steadily replaced by tissue that is progressing. Also, polylactic–polyglycolic acid copolymers and tricalcium phosphate [Ca_3_(PO_4_)_2_] are instances of this kind of biomaterials [11]. This review discussed the biocompatible material effects and their role in supporting dental implant prostheses.

## 2. The Role of Biomaterial in Prosthesis Implant

Using dental implants has been widely accepted as a common method of repairing dentition defects over the past decades. Dental implants are simply infected because of the oral pathogenic bacteria, although the rate of implant survival is enhanced to about 95 ± 2 percent over a 10-year follow-up duration. Occlusal overload and oral biofilms are two principle etiologies of peri-implantitis, and oral biofilms that have progress on dental prostheses have a vital role in the pathogenesis of peri-implantitis. In the absence of therapy and prevention, implant loss occurred due to peri-implantitis. The implant can be connected with oral bacterial cells, blood, and saliva during and after the surgery of implantation, and bacterial cells are linked to the abutment harm of encompassing gingiva [12]. The chemical and physical features of dental prostheses and materials are restorative and can affect pellicle coating, formation of biofilm, and adhesion of initial bacteria. Dental material application is progressing and has shown accelerating necessity to better find out the responses between the surface material and biofilm in the oral cavity. A higher range of biofilm density and viable microbial cells on the structure of prostheses based on Co–Cr (cobalt-chromium) alloys was detected, when contrasted with prostheses attributed to titanium, for the base-metal alloys [13]. One of the gold standards for oral implants is titanium implant screws because of their facility and exceeding biocompatibility to achieve osseointegration. The anticipated hypothesis is attaining a straight connection between the implant and living bone to ensure the long-standing action of the fixed prosthetic instrument [14].

## 3. The Role of Biomaterial in Surface Modifications and Coatings

Dental implant surface modification has been named as a valuable approach to support osseointegration and also facilitate the correlation between cells and biological fluids to accelerate the regeneration of peri-implant bone. Recently, different surface modification methods have been suggested and examined to develop the osseointegration of implants. One of the most common surface modifications, used in modern dental prostheses, is microroughness. Moreover, it has been known as a crucial function in linking to nearby tissues and anchoring cells which are appropriate for peri-implant osteogenesis. Various physicochemical approaches have been progressed to identify the roughness of implant surface, for example, acid-etching, grit-blasting, or combinations. Grit-blasting is usually conducted by hydroxyapatite, TiO_2_ particles, silica, and alumina. Acid-etching is performing as a homogenizer of the implant surface microprofile and eliminating the remaining blasting particles. Sulfuric acid, nitric, hydrofluoric, or combinations are the chemical agents which are used for acid-etching [15].

## 4. The Role of Biocompatibility in Healing of Dental Prosthesis

Nonosteogenic cells contribute to the early stage of healing procedures and are called to prescribe definite following phases in process of healing which is regulated by osteogenic cells. Macrophage cells are conducted as an important character in the initial phases of the process of bone healing implants due to macrophage-controlled immunoinflammatory reaction to the implanted materials and their effect on the result of healing response of the osteogenic cells. Therefore, the primary function of macrophage cells after implantation influences the result of bone healing and identifies the quality of tissue healing procedures. Regenerative macrophage phenotype is expressed dominantly and also crucially correlated with the healing of favor bone through osteogenic cell differentiation and producing different types of growth factors and cytokines [16]. In recent years, most studies are concerned with the healing of soft tissue surrounding implants and the integration of hard tissue of dental implants because of their central character in long-term maintenance. Also, it has been displayed that concentrates of platelet have a particularly pronounced impact on wound healing of soft tissue in comparison with hard tissue because of their attributes with different growth factors containing TGF-*β*1 (transforming growth factor-*β*1), VEGF (vascular endothelial growth factor), and PDGF (platelet-derived growth factor). Marx et al. use PRP (platelet-rich plasma) in dental cases; afterward, the utilization of PRP is broadly approved in various fields of orthopedics, esthetics, and dentistry in tissue regeneration due to their potential in enhancing angiogenesis. Moreover, they have limitations because of their contribution with anticoagulants which are called suppressors of regeneration in tissues. Therefore, PRF (platelet-rich fibrin) was developed to eliminate anticoagulant in 2001, and also PRF was used as a three-dimensional scaffold for tissue regeneration that has various advantages such as quick tissues angiogenesis, faster wound healing, and complete immune-biocompatibility [17].

## 5. The Role of Materials in Antibacterial and Anti-Inflammatory Effects

Various types of dental implant surface coating including fluoride, copper, chlorhexidine, zinc, silver, and antibiotics (e.g., amoxicillin, gentamycin) have been examined to supply antibacterial effects [18]. Infections surrounding implant instruments are related to biofilm and microbial infections that are linked to a solid surface. These microbial infections are exceedingly complex to treat bacteria that are adsorbed and resistant to immune system mechanisms and antimicrobials. Biomaterial surface has various properties such as hydrophobicity, roughness, charge, and micro- and nanostructure which have an important role in preventing biofilm infection on an implant surface. Lesser biofilm susceptibility to antimicrobial agents with diverse resistance of antibiotics for many bacteria strains requires study development on novel options for antibacterial approaches [19]. Some implant surfaces have anti-inflammatory and antibacterial effects with immobilization of bioactive molecules such as peptides, proteins, and growth factors. Nevertheless, using bioactive molecules has some disadvantages like short half-life, deficient stability, exorbitant cost, and side effects. To provide appropriate bioactive surfaces that may be easily converted to clinical applications, ZnO and Ag nanoparticles as metal nanoparticles have been examined because of their ability as anti-inflammatory and antibacterial agents [20]. Titanium bacterial colonization leads to the loss of the implant due to a formation of biofilm which is considered to help bacteria escape antibiotics and the host defense mechanism. Pathogens cause loss of bone surrounding implant; thus, it is necessary to do surgery; either the influenced bone is damaged or infected implants are removed or replaced. The infected locations are also treated with systemic antibiotics to eliminate the existence of bacteria. Consequently, antimicrobial agents such as sliver and fluorine ions are necessary to contribute to the dental implant to bind to the inside proteins of the bacteria for inhibiting the activities. Also, these ions are integrated on the titanium surface and have been exhibited to be useful against the formation of bacterial biofilm. Coatings of antibiotic releasing, made of fixed antimicrobial oligopeptides, are helpful in a short duration and may not prevent peri-implantitis after years of implantation. Using polymer-based materials with antibacterial features is another choice. Antimicrobial features are based on their structure as a consequence of organic or inorganic antimicrobial agents' introduction, as a consequence of the chemical modification. Chitosan is one of the biocompatible and antibacterial materials that also prevent the action of pathogens. Although the antibacterial mechanism of chitosan is not clear, it is considered the positive charge of amines' captivate negative charge of bacteria cell walls due to cell membrane disruption or the cell dynamics [21].

## 6. The Role of Materials in Osseointegration

Osseointegration has also been known as a dynamic process in which initial stability is replaced by secondary stability. Instantly after installation of implants, primary stability prepares mechanical stabilization by direct connection between the dental implant surface and the surface of the bony wall of the implant bed. It has been shown that the surface properties of biomaterials such as titanium dental implants have a decisive effect on the rate of osseointegration. Recently, dental implants made of zirconia and titanium alloys have been examined as an alternative biomaterial to replace missing teeth. Titanium alloys such as TiZr (titanium-zirconium) and Ti6Al4V (titanium-6aluminum-4vanadium) have better mechanical features than zirconia, and pure grade 4 titanium or ceramics compounds possess more advantages than titanium alloys and titanium. Surface modifications of these biomaterials eventually have effects on the procedures of osseointegration [22]. As a result of the chemical and physical connection between the surface of implants and the bone tissue, osseointegration occurred. The bone-implant interface has an exceedingly dynamic structure in which oxidative stress resulting from implant surgical insertion to the bone causes the surface TiO_2_ layer thickening which integrates phosphorus and calcium ions from the bone matrix [23]. Bioactivity has displayed automatic carbonated HA (hydroxyapatite) layer formation on the surface of biomaterial after its absorbance to the body fluid. Conversely, the HA layer that is formed can promote powerful bonding to the bone as a result of osseointegration [24].

## 7. The Role of Materials in Dental Pulp Regeneration

Dental pulp regeneration has been named as a challenging and complicated system that depends on vascularization and reinforced tissue. Endodontic regeneration is composed of regeneration of connective tissue and pulp, revascularization, and dentin formation [25]. Biomaterial designing is also related to the controlled release of molecular signaling of bioactive materials and induces mesenchymal stem cell differentiation to odontoblasts as a high-potential method in comparison with conventional endodontic therapy [26]. Chitosan scaffolds were loaded with biomolecules and growth factors because of increment of odontoblastic marker expression like alkaline phosphate, dentin matrix acidic phosphoprotein, and dentin sialophosphoprotein, preparing an extracellular matrix-like environment for differentiation and proliferation of dental pulp cells to the odontoblasts with biomineralization capacity [27]. Moreover, chitosan-based scaffolds as a novel biomaterial including molecular signaling such as mineral contents, BMPs (bone morphogenetic proteins), and drugs (i.e., metformin and simvastatin) have been stated to induce adhesion of cells, proliferation, and differentiation of dental pulp stem cells. TGF-*β*1 is an important biomolecule concerned with critical pulp therapy since it may be conducted as a regulator of the activity of alkaline phosphatase, the induction of the odontoblast-like cell proliferation, expression of OCN gene/protein, and mineral deposition [28]. Rehabilitation of dentinopulpal defect is one of the long-term complications in dentistry. Various restrictions of biomaterials are utilized as scaffolds for complicated regeneration of dentin pulp or used in restorative dentistry to stimulate dentin to seal the exposed pulp chamber. Also, the procedure of regeneration or reparation sometimes might be uncompleted [29].

## 8. Common Biocompatible Material in Supported Dental Implants

Biocompatibility has been determined as the compatibility of the material based on the biological environment. A long-standing connection between particular functions and tissues has an important role in dental implantation. Furthermore, biocompatibility has been defined with responses between implants and tissue examination which are examined in studies (*in vivo* or *in vitro*) [11]. Additionally, biocompatible materials that are utilized as dental implants may be categorized as bioactive, bioinert, and biotolerant [30]. The common biocompatible materials are mentioned in Table 1.

### 8.1. Bioactive Glass

Bioactive glass (BG) is one of the biomaterials which are used currently. Bioactive materials are associated with the biological conditions to evoke a particular response like the hydroxyapatite layer forming with a formation of the bond between biomaterials and tissues. Dentin, enamel, teeth, and bone are mainly composed of hard mineral tissue in the structure of crystalline calcium phosphate and hydroxyapatite. On the contrary, bioinert materials repress any reactions or communication of the biological environment. Nevertheless, these biomaterials affect environmental responses and fibrous capsule formation. The fibrous capsule can cause the prosthesis to micromovement and failures. Also, bioactive materials can be osteoinductive or osteoconductive, and their abilities indicate BG applications in abundant clinical situations comprising hard tissue regeneration in dentistry and medicine. BG is commonly used as coating material of dental implants, mineralizing agents, treatment of root canal, pulp capping, air-abrasion, and dental rehabilitation materials in dentistry. In medicine, it uses several applications from the restoration of soft tissue to orthopedics [40].

### 8.2. Collagen

One of the most common biomaterials used in the process of implantation and dental therapy is collagen. Collagen is also utilized in the different pathways as being prepared to linked cross-link or utilized in films, composites, three-dimensional matrix, and lattice-like gel. Also, collagen can improve restoration and granulation of tissues, protects wounds and tissues from infections mechanically, and has analgesic effects [41, 42]. Hence, hydrolyzed collagen can be utilized as a healing process booster, binding tissue fluids, and is a compatible biomaterial in dental therapy. Moreover, collagen not only has an important impact on the rejuvenation of epithelial cells but also is nontoxic, biodegradable, and well absorbed. For example, collagen membrane-scaffold graft in combination with recombinant human platelet-derived growth factor facilitates both regeneration and fibroblast adhesion to connective tissue. Therefore, collagen is often combined with other biocompatible materials to enhance the quality and the rate of treatment of defects in dental implantation [43].

### 8.3. Chitosan

Chitin deacetylation provides a biomaterial called chitosan which has been examined with incorporative procedures for its applications. Chitosan has individual characteristics such as adhesion to mucose, nontoxicity, biodegradability, antifungal activity, antibacterial effects, and biocompatibility. Chitosan degradation, especially by lysosomes, does not make toxic compounds, and chitosan implantation does not promote the activity of the immune system. Chitosan-based scaffolds are commonly utilized for dentin pulp, periodontal, and bone regeneration in dentistry. Chitosan lacks mechanical application and bioactivity is necessary for cartilage and bone tissue engineering, although it is compatible with matching membrane characteristics. Moreover, chitosan scaffolds loaded with bioactive components, growth factors, and synthetic polymers have been examined to fix a composite material with increased mechanical features and facilitate osteogenesis [28]. Chitosan has an application in the bone generation of surrounding dental implants. Chitosan loaded with hDPSCs (human dental pulp stem cells) is implanted in rabbit models, and the findings demonstrated the osseointegration and healing of bone in comparison with xenografts in the animal models. Also, chitosan loaded with hDPSCs displayed its potential in the regeneration of bone formation surrounding dental implants and ameliorate osseointegration [44].

### 8.4. Polyhydroxyalkanoates

Various bacteria synthesize different biopolyesters like PHAs (polyhydroxyalkanoates) as storage materials for energy and intracellular carbon which have several applications in studies containing medical implants. These findings indicated that PHAs have many characteristics for appropriate implantation like compatibility of tissues, biodegradable properties, and adequate mechanical properties. Due to the hydrophobic features of PHAs, it is a favorable choice for encapsulating nanostructure or microparticle hydrophobic drugs [45].

### 8.5. Polyetheretherketone

PEEK (polyetheretherketone) has been commonly used in orthopedics and in clinical dentistry as a synthetic material in the coloring of teeth and other applications. For instance, PEEK has shown lower shielding of stress in comparison with titanium as dental implants because of suitable interactions between bone and PEEK. Thus, PEEK has the potential to use in immobilized and removable prostheses. Recently, researchers have also investigated the bioactivity of PEEK implants at the nanostructure. Regarding physical and mechanical features of PEEK that are similar to bone, PEEK has promising usage in various properties of dentistry. One of the important complications in utilizing PEEK as dental implants is improving the PEEK bioactivity without changing mechanical properties. Moreover, upgrading the properties of materials and modifications can increase its ability in dentistry [46].

### 8.6. Calcium Phosphate-Based

CaP (calcium phosphate) has been named due to the mineral including calcium ions with different types of phosphate and hydrogen or hydroxide ions. 100 years ago, CaP- (calcium phosphate-) based materials have been generally utilized in craniofacial surgery, and due to their outline potential, it is a candidate as a drug delivery system and bone substitutes. CPC (calcium phosphate cement) as a particular CaP biomaterial has favorable properties to use in coating implants that can facilitate the healing of bone surrounding implants. CaP-based materials also play an important role in dental implants because of their similarity to bone composition, their bioactive ability, and their osteoconductive properties. Also, CPC has various excellent capabilities to use as biomedical materials in clinical dentistry applications, which have great properties of bone repairing and tremendous biocompatibility [47].

### 8.7. Titanium

Ti is an element from transition metal (atomic number = 22) with lustrous silver color, high strength, and low density. Ti has high resistance to corrosion under different circumstances, and it is claimed that Ti has biocompatible and nontoxicity properties in humans. Therefore, Ti and Ti alloys have promising applications in several medical situations (i.e., implants and surgical implements) and clinical dentistry such as prostheses, abutment, and wires of orthodontic. Various materials such as stainless steel and Vitallium (cobalt-chromium) are candidates as an implant of the misplaced tooth, but the progression of technology and science of materials accepted that Ti becomes the pioneer and the most common biocompatible material because of its properties. Ti is broadly successful in the process of implantation because of its abundant advantages. Ti is a bioinert material that can bind to osteoblasts with its great biocompatibility. Ti oxides have high stability and suitable resistance against corrosion as film and may divide the bulk titanium from the surrounding parts [48]. Because of the compact Ti oxide thin layer in the surface, Ti and its alloys have excellent biocompatibility and favorable metallic selection for subgingival implants and are also utilized as coatings and surface modification to stimulate osseointegration. Titanium and its alloys have tremendous abilities to bind to bone and living tissue. Usually, metal ions such as Co, Hg, Cr, Cu, Zn, and Sn that are located on a culture medium can induce inhibited zone of different organisms which demonstrated damage to cells and cytotoxicity. Also, these metal ions and their alloys are used in clinical dentistry as components and constituents for dental amalgams. Ti alloys, such as TiMo and TiNi, are utilized as materials in the wire of orthodontics because of their memory characteristics and particular spring [5].

### 8.8. Au

Au (gold) has been known as dental prostheses since a long time ago, because it has resistance to corrosion, has an appropriate melting point, and might attain suitable mechanical features by alloying. Gold like copper and silver fixes Ti b-phase to a lower temperature by the diagram phase of binary equilibrium. Ti_3_Au forms as an intermetallic compound contain gold with a concentration of about 15%. Thus, gold is a favorable alloying element for Ti with positive influences on the grinding capability and mechanical characteristics. Passive layers of Ti-alloy include metal defect, outer, intermediate, and inner layers. The Ti-Au outer layer makes primary galvanic corrosion. The current density of initial galvanic corrosion is decreased due to the porous and thin outermost layer which is formed [48, 49].

### 8.9. Ceramic

Ceramic is originally based on “keramos” which is a Greek word and is definite as burnt or pottery article. Nowadays, ceramic has various wide definitions and contains cement systems, advanced ceramics, and glass. Also, ceramics such as nonmetallic and inorganic solids are commonly produced with adequate heat approaches and then cooling, which are seldom regarded as covalent combinations and ionic metallic bonding. Materials of ceramics can be noncrystalline, partly crystalline, and crystalline which consisted of glass or pure ceramics. YSTZP (Yttria-stabilized tetragonal polycrystals of zirconia) is one of the most frequent ceramics which is used as the dental implant due to its toughness formation and appropriate biocompatibility property. Nevertheless, the stability of YSTZP induced by degradation of temperature and stress can be useful in protection and improve the strength of integration between tissue and implant. During production, many fabrication procedures in the industry can lead to final microstructures of YSTZP material that commonly have different stability [1]. ZBC (ZrO2-based ceramics) is conducted as an important material in medical instrument applications. Recently, utilization of ZBC has been significantly increased as a biomaterial of dental crowns and implants because of ZBC's excellent mechanical properties such as biocompatibility, strengths, and great resistance to friction and abrasion. Zirconia has been named as a promising ceramic material for medical device application particularly in clinical dentistry because of corrosion resistance, effective biocompatibility, and low weight features in comparison with other ceramics. One of the explicit advantages of ZBC over Ti or the other metallic implants is being a natural choice to generate immobilized teeth substitutes [50].

### 8.10. Polymer-Based Implants

Polymer-based implants are comprised of polymeric materials such as PET (polyethylene terephthalate), PEEK (polyether ether ketone), PTFE (polytetrafluoroethylene), PU (polyurethane), PMMA (polymethylmethacrylate), PE (polyethylene), UHMW-PE (ultra-high-molecular-weight polyethylene), and PDS (polydimethylsiloxane) which have been used for replacement of missing teeth and dental roots [11]. Materials with major similarity to the color of teeth and bone, for instance, elasticity modulus, are examined to be used in implantation such as a composite of fiber‐reinforced PEEK and alternative to Ti. PEEK is also used in dental abutments, implants, and mobilized and fixed prostheses. The hypothetical benefits of PEEK in Young's modulus indicated that PEEK is similar to bone in comparison with Ti, particularly ceramic implants, but these trials just investigated in silico simulation, and long-standing clinical studies might be examined and confirmed [1]. Generally, polymer-based materials prepare properties in dental implant and root progressions such as excellent porosity, electrical and thermal apathy, biocompatibility, elastic modulus that is like soft tissue, simple controlling, low-priced fabrication, and suitable stretching in contrast to other biocompatible materials. Nevertheless, polymer-based implants are more complicated for sterilization with autoclave or ethylene oxide. Electrostatic responses of surfaces of polymers and the cleaning condition of oral environments may collect micro- and macroparticulates. The open zones of tissues that lead to growth are closed by deforming porous polymers which depend on elasticity [11].

## 9. Future Challenges

Recently, the knowledge about biocompatible materials and biomolecular-related procedures has been developed and displayed the progression in repairmen and regeneration of tissues that are linked to teeth, but several experiments have remained to find out the potential properties of biocompatible materials. Also, functional models of biomaterials can prepare favorable esthetics, inhibit the formation of biofilm, and stimulate remineralization and some prospective challenges for clinical approaches. Accelerating clinical translation procedures and preparing powerful fabrication, approvable testing data and methods are accessible. Thus, these abilities can help in the findings of new biomaterials which are needed [51].

## 10. Conclusion

In recent decades, many biocompatible materials are used in dental implants and prostheses due to their ability to have anti-inflammatory properties. Physicochemical modifications of dental prostheses also reduce the adhesion of microorganisms but cannot inhibit peri-implantitis. Biocompatible materials have been commonly used as antimicrobial agents to sustain the treatment and prevention of peri-implantitis and mucositis of peri-implant. Also, some types of biocompatible materials such as polymer-based implants, metal implants, and natural bioactive materials indicate their role in the wound and bone healing process, stimulating dental pulp regeneration and surface modification of dental implants. Further researches are needed to achieve the ideal dental implant.

## Figures and Tables

**Table 1:** The common biocompatible materials as supportive substances in implantation of dental prosthesis.

Type	Design	Method	Result	Ref/year
Carbon-reinforced polyether ether ketone (CRF-PEEK)	*In vitro*	3D (three-dimensional) model of implants in the first mandibular molar using a combination of lithium disilicate, Ti, and CRF-PEEK for abutment/implant materials	Replacement of Ti implant with PEEK implant does not prepare significant advantages to lead to improved stress distribution to the bone with peri-implant	[31]/(2019)

Hydroxyapatite (HA)	*In vivo*	Ti implants with HA coatings and grit-blasted surfaces (Al_2_O_3_) as control were embedded in rabbit tibiae	Coating with HA can improve the physical and chemical properties of osseointegration in comparison with the grit-blasted implant	[32]/(2019)

Polyetheretherketone (PEEK)	*In vitro*	Investigation of bioactivity in PEEK sample coated with pure Ti by electron beam deposition technique and unmodified PEEK sample	Dental implants with PEEK and electron beam deposition of Ti as surface modification have increased bioactivity in comparison with unmodified PEEK implants	[33]/(2020)

Chitosan	*In vitro*	Evaluation of the cell viability, morphology, and osteogenic capacity of chitosan in dental implants	The combination of chitosan and laser surface increases the healing process and osseointegration of dental implants	[34]/(2020)

Polymethylmethacrylate (PMMA)	*In vivo*	Investigation of PMMA-based material in patients with compromised dentitions as immobilized dental prostheses	PMMA-based material can be utilized with 3- to 4-unit FDPs for long-term temporization at least more than a year	[35]/(2014)

Ceramic	*In vivo*	Investigation of MC (metal-based ceramic) and ZC (zirconia-based ceramic) in posterior immobilized dental prostheses in patients	MC and ZC posterior immobilized dental prostheses have the same results as the most results measured in 10 years	[36]/(2018)

Keratinized tissues (KT)	*In vivo*	Evaluation of the influence of the width of KT on peri-implant tissues by investigation of peri-implant clinical and inflammatory parameters	Free gingival graft around KT causes major improvements in inflammatory parameters and peri-implant clinical features	[37]/(2013)

Titanium (Ti)/Zirconia (Zr)	*In vivo*	Evaluation of proinflammatory cytokines and mediators of bone metabolism in patients who received Ti and Zr as abutments fixed dental implants	Ti and Zr as abutment biomaterials have no remarkable difference in mediator profiles of bone metabolism and proinflammatory cytokines	[38]/(2015)

Chitosan-enriched fibrin hydrogel	*In vitro*	Investigation of antimicrobial influences (with growth analysis of *Enterococcus faecalis*), DP-MSC (dental pulp-mesenchymal stem/stromal cell) viability, proliferation, production of collagen, and deposition of extracellular matrix	Chitosan-enriched fibrin hydrogel can stimulate neoformation of human dental pulp tissue due to the antimicrobial effects of chitosan and influence on the morphology of dental pulp cell, proliferation, viability, and production of collagenous matrix	[39]/(2019)

## Data Availability

This article is a review and does not contain any studies with humans or animals performed by any of the authors.
